# Reaction of *cis*- and *trans*-Dichlorotetra(Dimethylsulfoxide)Ruthenium(II) With the Antiviral Drug Acyclovir

**DOI:** 10.1155/MBD.2000.325

**Published:** 2000

**Authors:** Aglaia Koutsodimou, Giovanni Natile

**Affiliations:** 1 NCSR Demokritos Institute of Physical Chemistry Paraskevi Attikis GR-153 10 Ag Greece; 2 Dipartimento Farmaco-Chimico via E. Orabona 4 Bari I-70125 Italy

## Abstract

NMR was used to investigate the reaction of *cis*- and *trans*-[RuCl_2_(DMSO)_4_] with the antiviral drug acyclovir, a guanine derivative containing the acyclic (2-hydroxo) ethoxymethyl pendant linked to N(9). Studies were performed in aqueous solutions at ambient temperature and at 37 ^°^C, and at various molar ratios. Both isomers yielded two compounds, a monoadduct and a bisadduct, the relative yields being dependent upon the metal to ligand concentration ratios. The products derived from the two Ru isomers displayed identical NMR spectra, suggesting that they have the same coordination environment, however the rate of formation of the monoadduct was higher in the case of the *trans* isomer than in the case of the *cis* isomer, while the rate of conversion of the monoadduct into the bisadduct appeared to be similar in both cases. As a consequence in the case of the *trans* isomer there is accumulation of monoadduct in the early stage of the reaction, whose concentration afterwards decreases with the progress of the reaction. As for platinum, also for ruthenium the preferred binding site is N(7) of the purine base, however, in the case of ruthenium a discrete amount of bisadduct is formed even in the presence of an excess of metallic substrate with respect to the acyclovir ligand; under similar conditions a platinum substrate would have given, nearly exclusively, the monoadduct.


### REACTION OF *cis*- AND *trans*-DICHLOROTETRA(DIMETHYLSULFOXIDE)RUTHENIUM(II) WITH THE ANTIVIRAL DRUG ACYCLOVIR

Aglaia Koutsodimou[1] and Giovanni Natile*[2]

[1] NCSR Demokritos, Institute of Physical Chemistry, GR-153 10 Ag. Paraskevi Attikis, Greece. <lkoutsod@mail.demokritos.gr>
[2] Dipartimento Farmaco-Chimico, via E. Orabona 4, I-70125 Bari, Italy. < natile@farmchim.uniba.it>

### ABSTRACT

NMR was used to investigate the reaction of *cis*- and *trans*-[$RuCl_2(DMSO)_4$] with the antiviral drug acyclovir, a guanine derivative containing the acyclic (2-hydroxo)ethoxymethyl pendant linked to N(9). Studies were performed in aqueous solutions at ambient temperature and at 37 ° C, and at various molar ratios. Both isomers yielded two compounds, a monoadduct and a bisadduct, the relative yields being dependent upon the metal to ligand concentration ratios. The products derived from the two Ru isomers displayed identical NMR spectra, suggesting that they have the same coordination environment, however the rate of formation of the monoadduct was higher in the case of the *trans* isomer than in the case of the *cis* isomer, while the rate of conversion of the monoadduct into the bisadduct appeared to be similar in both cases. As a consequence in the case of the *trans* isomer there is accumulation of monoadduct in the early stage of the reaction, whose concentration afterwards decreases with the progress of the reaction. As for platinum, also for ruthenium the preferred binding site is N(7) of the purine base, however, in the case of ruthenium a discrete amount of bisadduct is formed even in the presence of an excess of metallic substrate with respect to the acyclovir ligand; under similar conditions a platinum substrate would have given, nearly exclusively, the monoadduct.

### INTRODUCTION

*Cisplatin*, *cis*-[$PtCl_2(NH_3)_2$], is one of the most widely used drugs in cancer therapy [1], however the appearance of severe side-effects, the drug resistance which develops after a prolonged treatment, and the limited spectrum of activity has led scientists to search for new anticancer metal based drugs. Ruthenium complexes are among those which have displayed a great potential for medical applications. In particular *cis*- and *trans*-[$RuCl_2(DMSO)_4$], two neutral octahedral Ru(II) compounds, possess significant antitumor activity and a remarkable antimetastatic activity in some murine tumor models [2-4]. The *cis* isomer has one *O*-bonded and three facial *S*-bonded DMSO molecules, while in the *trans* isomer, all four DMSO molecules are *S*-bonded. The *trans* isomer is markedly more toxic and reactive than the *cis* isomer [3, 5, 6]. *In vivo* and *in vitro* experiments showed that both isomers interact with DNA [7, 8] and preferential interaction with adjacent guanines was found upon treatment with pBR 322 DNA [9]. As for the platinum complexes, also for ruthenium the target atom of the guanine base appears to be N(7). Apart from the above mentioned compounds, also other ruthenium compounds have proved to be active antitumor drugs and one of them, NAMI-A, has entered clinical trials as an antimetastatic agent [10].

In previous papers we investigated the reaction of platinum compounds with the antiviral drug acyclovir [11-13]; a guanosine analog characterized by having an acyclic residue [a (2-hydroxy)ethoxymethyl group] linked to N(9). The scope was that of synthesizing multifunctional drugs in which either the anticancer activity of the platinum moiety, or the antiviral activity of the acyclovir residue could be enhanced. In some occasions interesting results, particularly regarding the antitumor activity, were obtained [14, 15].

A similar investigation has now been performed on two ruthenium substrates and the results are reported herein.

### MATERIALS AND METHODS

*Cis-* and *trans-*$[RuCl_2(DMSO)_4]$ were synthesized and recrystallized as previously reported [5, 16]. Acyclovir (Sigma) was used with no further purification. The $^1H$ (250.13 MHz) and $^{13}C$ NMR (62.90 MHz) spectra of the ligand and the complexes were obtained on a BRUKER WM-250 spectrometer in $D_2O$ (99.90% D) solutions using DSS as internal reference. The main parameters used for $^{13}C$ NMR were: 64k data domain size, 13889 Hz spectral width, 0.5s relaxation delay, and 28500 scans.

### RESULTS AND DISCUSSION

The chemical behavior of *cis-* and *trans-*$[RuCl_2(DMSO)_4]$ (abbreviated as *cis*-Ru and *trans*-Ru, respectively) in aqueous solutions has been extensively investigated [5, 7, 8]. Once dissolved in water the *cis* isomer releases its *O*-bonded DMSO molecule to give *cis,fac*-$[RuCl_2(H_2O)(DMSO)_3]$, which then slowly (in about 3h at 37 °C) substitutes a chloride ion with water, leading to *cis,fac*-$[RuCl(H_2O)_2(DMSO)_3]^+$. In contrast *trans*-$[RuCl_2(DMSO)_4]$ immediately replaces two neighboring DMSO molecules with water, generating *trans,cis,cis*-$[RuCl_2(H_2O)_2(DMSO)_2]$, which, slowly (about 8 h at 37 °C) exchanges one chlorine atom to give the cationic complex *fac,cis*-$[RuCl(H_2O)_3(DMSO)_2]^+$.

The reactions of *cis-* and *trans-*$[RuCl_2(DMSO)_4]$ with acyclovir (ACV, Scheme 1) were performed at different molar ratios (r = ACV:Ru = 1:1, 2:1, and 1:1.25), at room temperature and at 37 °C in unbuffered water solutions. The fully deuterated metal complexes were also used under identical experimental conditions in order to avoid overlapping of the methyl protons of the DMSO molecules with the proton resonances of the acyclovir alkyl groups.

NMR spectroscopy was employed both to characterize the metal complexes and to monitor the time course of the reaction.

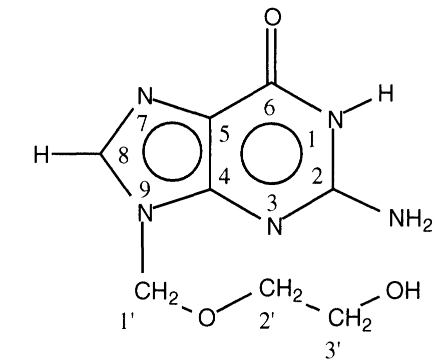

Scheme 1: Schematic drawing of ACV showing also the numbering scheme

### Reaction of *trans*-$[RuCl_2(DMSO)_4]$ with acyclovir

When *trans*-$[RuCl_2(DMSO)_4]$ reacts with an equimolar solution of acyclovir, at room temperature, two different reaction products, I and II, are formed. The time profile of the reaction is depicted in Fig. 1a . Complex I dominates during the first two hours of reaction. Integration of the H8 signals reveals that 60% of the overall species is present as complex I after 30 minutes. For longer reaction time complex I starts decreasing while a new product, compound II, continuously increases reaching its maximum concentration after 24 h. The free acyclovir concentration decreases dramatically during the first hour of reaction. In the final solution, no free acyclovir is detected whereas complex I accounts for *ca.* 40% of the reaction products and complex II for about 60%. Unreacted solvolysis products of *trans*-$[RuCl_2(DMSO)_4]$ are also present.

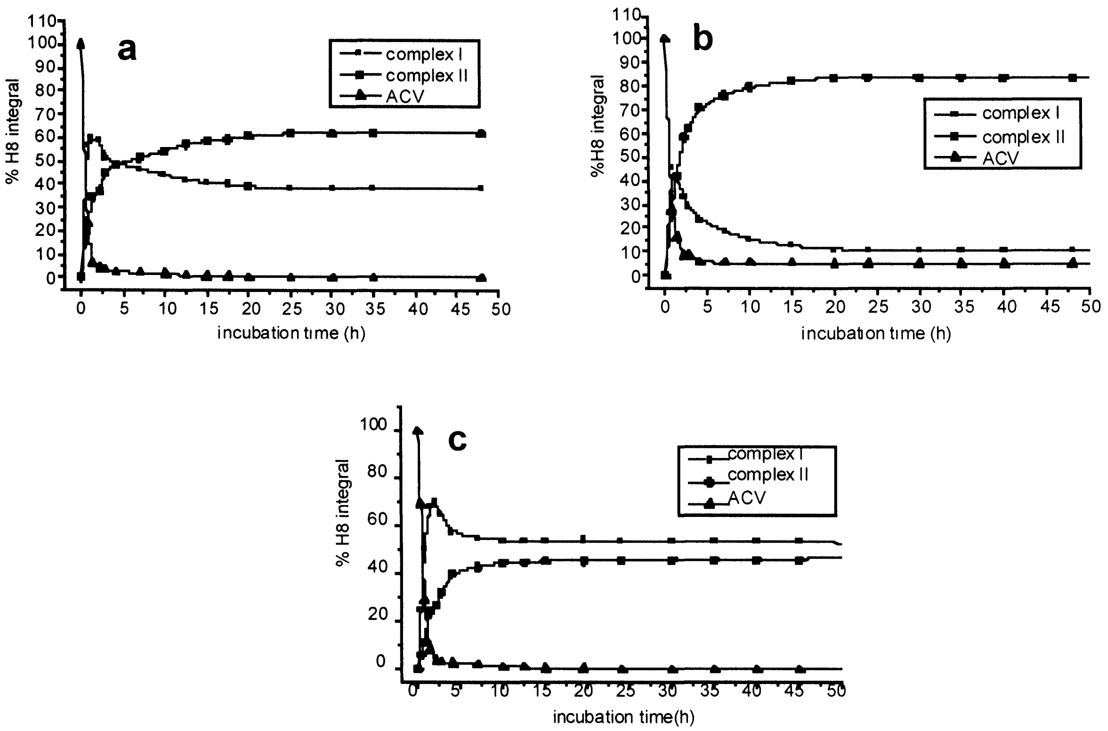

Figure 1. Time dependence of the concentration of different species in the reaction of *trans*-Ru with ACV (a, ACV:*trans*-Ru = 1:1; b, ACV:*trans*-Ru = 2:1; c, ACV:*trans*-Ru = 1:1.25) performed in water solution at room temperature. The concentrations were determined by integration of the peak areas of the aromatic region (H8 of ACV) in the $^1$H NMR spectra.

Using an acyclovir to *trans*-[$RuCl_2(DMSO)_4$] molar ratio of 2:1, the same products, compounds I and II, observed in the previous experiment are formed. However the time profile of the product formation (Fig. 1b) differs from that observed when using the reactants in a 1:1 molar ratio. The concentration of complex I again increases during the first 30 minutes, and then it starts decreasing. Complex II becomes the dominant species after 90 minutes (*ca.* 45%) and increases to *ca.* 70% of the overall reaction products after 4 hours. The solution reaches a steady composition within 24 h. Free acyclovir (8.5%) is present in the final reaction mixture in which 83% of total ACV is present as compound II, and 8.5% as compound I. All the *trans*-Ru substrate has been consumed by the end of the reaction.

The reaction progress observed using a slight excess of the ruthenium substrate (ACV:*trans*-Ru = r = 1:1,25) was very similar to that of r = 1 (Fig. 1c). However under these conditions the monoadduct remains the dominant species even at the end of the reaction (after 1 day). This seems reasonable since the ligand concentration is now even smaller than that of the Ru substrate. It is also worth noting that the percentage of bisadduct is still significantly high notwithstanding the excess of metallic substrate over the acyclovir ligand.

The reaction of acyclovir with *trans*-Ru has also been performed at 37 °C (Figure 2). It proceeds much faster (*ca* 10 times) than the corresponding reaction carried out at ambient temperature but the ratio of the products at the end of the reaction is similar.

In all the experiments performed with *trans*-Ru the final spectra exhibit two single resonances at $\delta$ = 8.85 and 8.58 ppm in the region of H8 resonances, belonging to compounds I and II, respectively (Fig. 3, Table I).

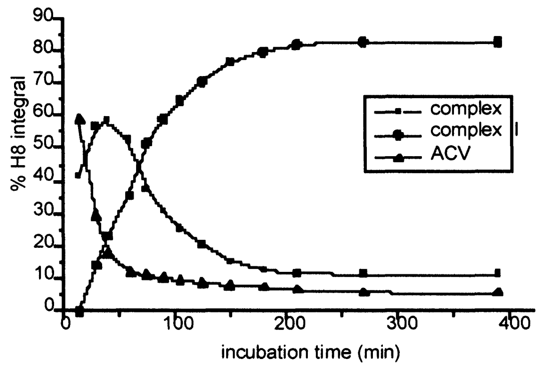

Figure 2. Time dependence of the concentration of different species in the reaction of *trans*-Ru with ACV (ACV:*trans*-Ru = 2:1) at 37 Æ in water solvent. The concentrations were determined by integration of the peak areas of the aromatic region (H8 of ACV) in the $^1$H NMR spectra.

The large downfield shifts of H8 (0,92 and 0,65 ppm for compounds I and II, respectively) are typical of N7 coordination of the guanine moiety to a transition metal center. This was previously observed for the complexes of *trans*-Ru with guanine bases. Specifically, the reaction of *trans*-Ru with 2'-dGuo [17, 18] leads to the formation of two diastereoisomeric monoadducts and a bisadduct, in which the guanosine is coordinated to Ru *via* N7. In the case of reaction of *trans*-Ru with 5'-dGMP [19] again two diastereoisomeric monoadducts are formed in which the nucleotide coordinates to the metal center through N(7) of the purine moiety and an oxygen of the phosphate group.

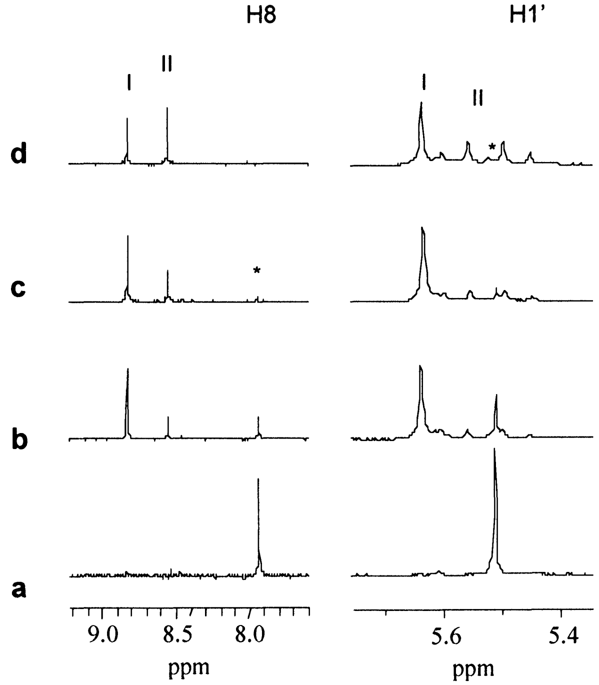

Figure 3. Sections of the $^1$H NMR spectra at different time intervals (a, 0 min; b, 40 min; c, 80 min; d, 24 h) of the reaction mixture ACV:*trans*-Ru = 1:1, at room temperature and $D_2O$ solvent. The peaks of unreacted ACV are denoted with an asterisk (*).

Table I: $^1$H NMR (δ, solvent $D_2O$) of ACV, hydrolyzed *trans*-Ru and *cis*-Ru, and their complexes.

| Compound | H(8) | H(1') | H(2') | H(3') | DMSO |
|---|---|---|---|---|---|
| ACV | 7.93 | 5.51 | 3.67 | 3.67 | |
| *trans*-Ru | | | | | |
| $[Ru(DMSO)_2Cl_2(H_2O)_2]$ | | | | | 3.35 |
| $[Ru(DMSO)_2Cl(H_2O)_3]^+$ | | | | | 3.40, 3.35 |
| *cis*-Ru | | | | | |
| $[Ru(DMSO)_3Cl_2(H_2O)]$ | | | | | 3.49, 3.47, 3.39 |
| $[Ru(DMSO)_3Cl(H_2O)_2]^+$ | | | | | 3.50, 3.43, 3.39 |
| Compound I | 8.85 | 5.66 | 3.73 | 3.73 | 3.44, 3.36,[a] 3.26 |
| Compound II | 8.58 | 5.55 m[b] | 3.49 m | 3.63 m | 3.35, 3.21 |

[a] The signal at 3.36 ppm, which however overlaps with the signal at 3.35 of compound II, appears to have twice the intensity of the signals at 3.44 and 3.26 ppm. [b] m = multiplet.

Reaction with d(GpG) leads to the formation of a stable compound with two N(7)-coordinated guanine moieties [20]. Bifunctional coordination was also found for the dinucleotides GpA, ApG, d(ApG) and d(GpA) [21] where the two purine bases are coordinated to ruthenium through N(7). The same coordination mode was observed for 9-ethylguanine (9-EtG) when reacted with Ru(II) compounds giving $[RuCl_2(\eta^6\text{-}C_6H_6)(9\text{-}EtG)]$ and *cis*-$[Ru(dpy)_2(9\text{-}EtG)L]^{n+}$ (dpy = 2,2'-dipyridyl, n = 1 or 2 for L = $Cl^-$ or $H_2O$, respectively) [22, 23].

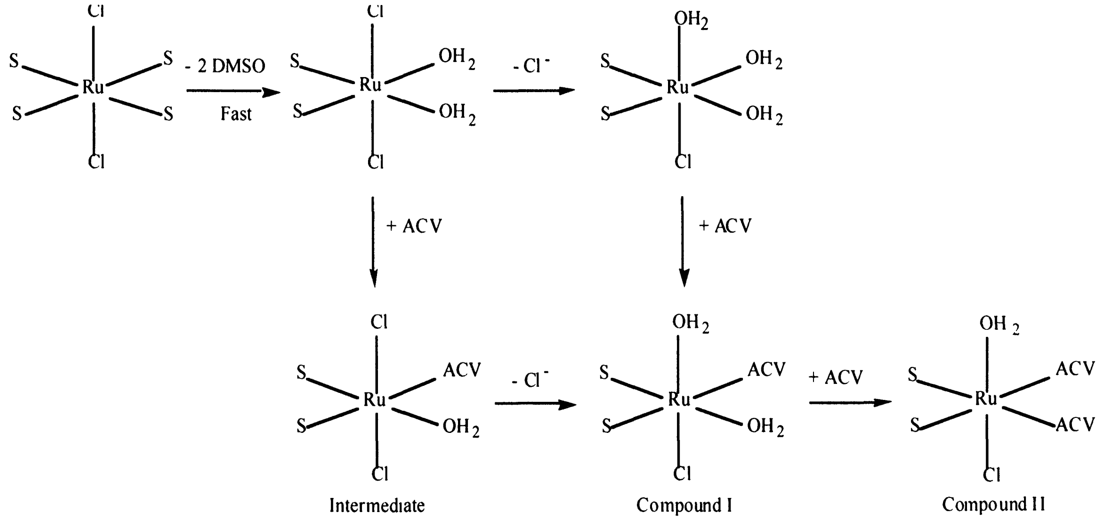

Scheme2. Proposed mechanism for the reaction of *trans*-$[RuCl_2(DMSO)_4]$ with ACV in water solution. S = *S*-bonded DMSO. The overall charge of the complexes is not indicated, it is 0 for species containing two chlorine ligands and +1 for species containing only one chlorine ligand.

The H(1') protons of the monoadduct appear as a singlet at $\delta = 5.66$ ppm (Fig. 3). In the same region (5.57 ppm) the H(1') atoms of the bisadduct appear as an AB quartet indicating that the C(1')$H_2$ protons are not equivalent but undergo a diastereotopic splitting of *ca.* 0.1 ppm. The non-equivalence of the C(1')$H_2$ protons is characteristic of a bisadduct with the two ACV ligands in cis positions and can originate from a restricted rotation of the alkyl chain about the C(1')-N(9) bond and of the two coordinated acyclovir molecules about the N(7)-Ru bonds. The H(2') and H(3') protons also appear as a singlet in the monoadduct and as multiplets in the bisadduct.

The DMSO methyl signals partially overlap with the H(2') and H(3') resonances of the disubstituted compound. In order to identify unequivocally the peaks of the DMSO ligands the spectra were compared with those obtained using the fully deuterated *trans*-Ru species. On this ground the two equally intense resonances at 3.42 and 3.25 ppm and a resonance at 3.36 ppm, partially overlapping with one signal of compound II and having an intensity twice the intensity of the signals at 3.44 and 3.26 ppm, (Table I) were assigned to the DMSO methyl groups belonging to the monoadduct, while another set of two equally intense signals at 3.20 and 3.34 ppm was assigned to the DMSO methyl protons of the bisadduct. Evidence that the DMSO ligands are bound through the S atom is provided by the fact that there is a small difference in the chemical shifts of the DMSO protons of the hydrolyzed *trans*-Ru species and those of compounds I and II.

In addition to the complexed species, also the methyl signals of hydrolyzed *trans*-Ru species, *trans,cis,cis*-$[RuCl_2(H_2O)_2(DMSO)_2]$ (3.35 ppm) and *fac,cis*-$[RuCl(H_2O)_3(DMSO)_2]^+$ (3.38 and 3.37 ppm), and of free DMSO (2.72 ppm) were observed in the cases of r = 1:1 and 1:1.25.

The assignments of the $^{13}C$ NMR spectra of the two compounds are reported in Table II. A considerable downfield shift of C(8) (monoadduct +4.8, bisadduct +4.2 ppm) confirms the formation of Ru-N(7) bonds. Negligible shifts were observed for the alkyl chain carbons, C(1'), C(2') and C(3'), confirming the non involvement in the metal coordination of either the ether oxygen or the hydroxyl group.

Table II: $^{13}C$ NMR ($\delta$, solvent $D_2O$) of ACV, hydrolyzed *trans*-Ru, and their complexes.

| Compound | C(6) | C(2) | C(4) | C(8) | C(5) | C(1') | C(2') | C(3') | DMSO |
|---|---|---|---|---|---|---|---|---|---|
| ACV (in $D_2O$) | | | | 141.9 | | 74.8 | 72.2 | 62.3 | |
| ACV (in DMSO-$d_6$)[a] | 160.8 | 157.8 | 155.4 | 141.7 | 120.4 | 76.0 | 74.4 | 63.9 | |
| *trans*-Ru | | | | | | | | | |
| $[Ru(DMSO)_2Cl_2(H_2O)_2]$ | | | | | | | | | 44.6 |
| | | | | | | | | | 44.0 |
| $[Ru(DMSO)_2Cl(H_2O)_3]^+$ | | | | | | | | | 45.8 |
| | | | | | | | | | 45.6 |
| Compound I | 160.4 | 156.0 | 154.9 | 146.7 | 117.5 | 74.3 | 72.1 | 61.6 | 46.2 |
| | | | | | | | | | 45.8 |
| Compound II | 159.9 | 155.9 | 154.6 | 146.1 | 117.2 | 74.7 | 71.8 | 61.5 | 45.2 |
| | | | | | | | | | 45.0 |

[a] From reference 28

The DMSO methyl peaks in the $^{13}C$ NMR spectra also confirm the findings of the $^1H$ NMR spectra. Two resonances were observed for each one of the reaction products. The difference in chemical shifts were comparable to that observed for the two carbon resonances of the cation *fac,cis*-$[RuCl(H_2O)_3(DMSO)_2]^+$.

**Reaction of *cis*-$[RuCl_2(DMSO)_4]$ with acyclovir**

The reaction of *cis*-$[RuCl_2(DMSO)_4]$ with acyclovir, in water solution, was monitored by NMR and the appearance of the different species as a function of time is depicted in Fig. 4. Two products are formed also in this case, they exhibit $^1H$ NMR spectra identical to those of the compounds obtained in the previous reaction of the *trans*-Ru isomer with ACV.

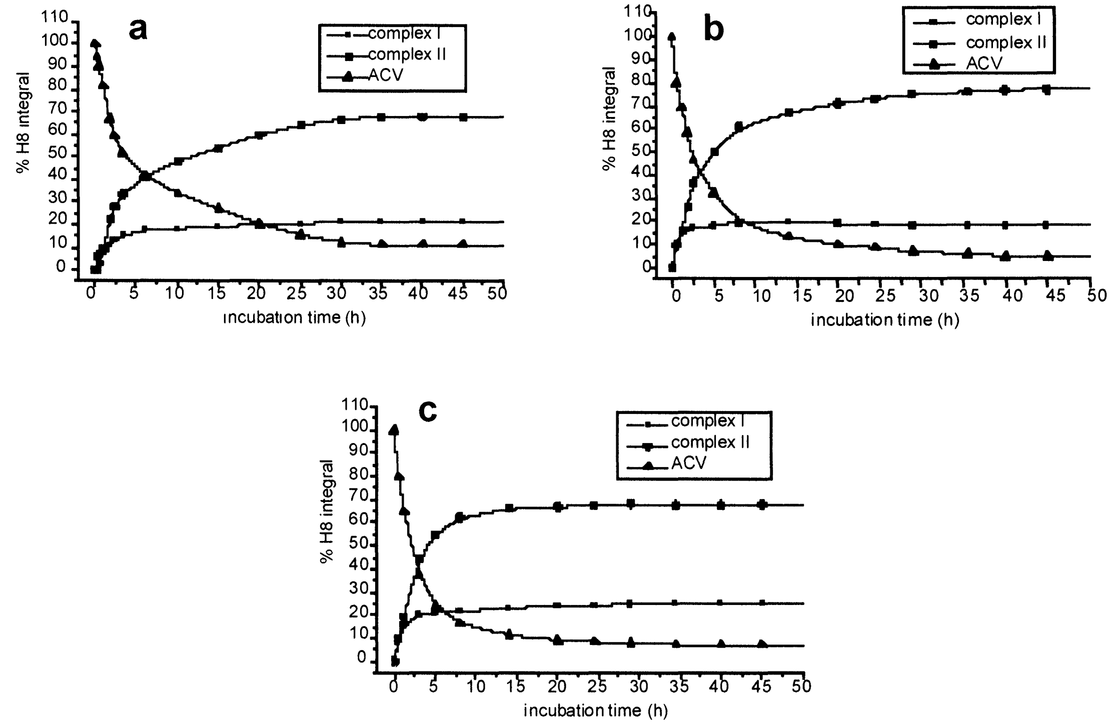

Figure 4. Time dependence of the concentration of different species in the reaction of *cis*-Ru with ACV (a, ACV:*cis*-Ru = 1:1; b, ACV:*cis*-Ru = 2:1; c, ACV:*cis*-Ru = 1:1.25) performed in water solution at room temperature. The concentrations were determined by integration of the peak areas of the aromatic region (H8 of ACV) in the $^1$H NMR spectra.

The kinetic profiles observed in the reactions of *cis*-Ru with acyclovir differ from those observed for the *trans*-Ru isomer. When the reactions are carried on at room temperature the solution reaches a steady composition after 40 - 50 hour reaction time. In all cases the bisadduct is the compound that dominates after the first hour of reaction. The concentration of the monoadduct increases during the first 5 hours reaching about 20% of the overall ACV species present in solution. At the end of the reaction the bisadduct accounts for about 70% of the total for r = 1:1 or 1:1.25, while at a molar ratio of 2:1 it reaches about 80% of the total ACV species.

The reaction of acyclovir with *cis*-Ru has also been recorded at 37 _C for r = 1 and 2. The rates are much faster (*ca.* 10 times) but the type and concentration of products are the same as those observed at ambient temperature.

It is worth noting that in all the reactions performed with the *cis*-Ru complex, the concentration of complex I in the initial stage of the reaction never increases above that at the end of the reaction, contrary to what has been observed in all reactions performed with *trans*-Ru (Fig. 1a-1c) where the concentration of complex I reaches a maximum in the early stage of the reaction and then decreases.

As mentioned earlier, the $^1$H NMR spectra of the two reaction products, (compounds I and II) obtained in the reaction of acyclovir with *cis*-Ru are exactly the same as those obtained in the reaction of acyclovir with *trans*-Ru. However, during the progress of the reaction, the DMSO methyl region is different, reflecting the different hydrolysis products of *cis*-Ru as compared to those of *trans*-Ru [5, 16]. Fig. 5 reports the reaction progress in the case of ambient temperature and using a slight excess of metal substrate (r = 1:1.25).

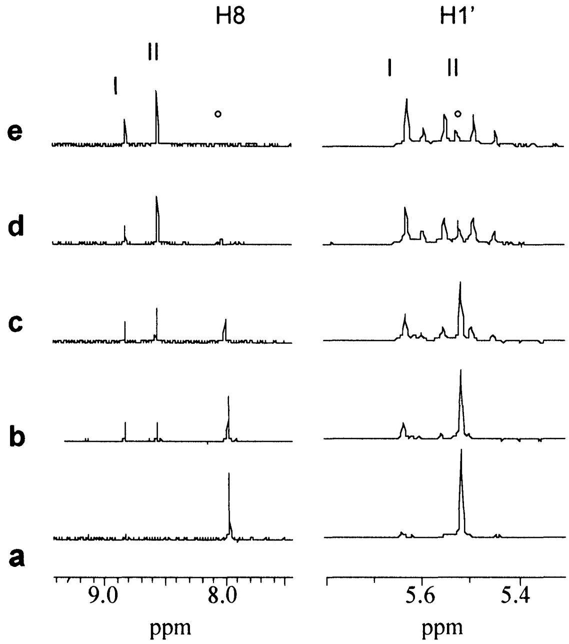

Figure 5. Sections of the $^1$H NMR spectra at different time intervals (a, 0 h; b, 1 h; c, 2,5 h, d, 8 h; e, 29 h) of the reaction mixture ACV:*cis*-Ru = 1:1.25, at room temperature and $D_2O$ solvent. The peaks of unreacted ACV are indicated with a circle (°).

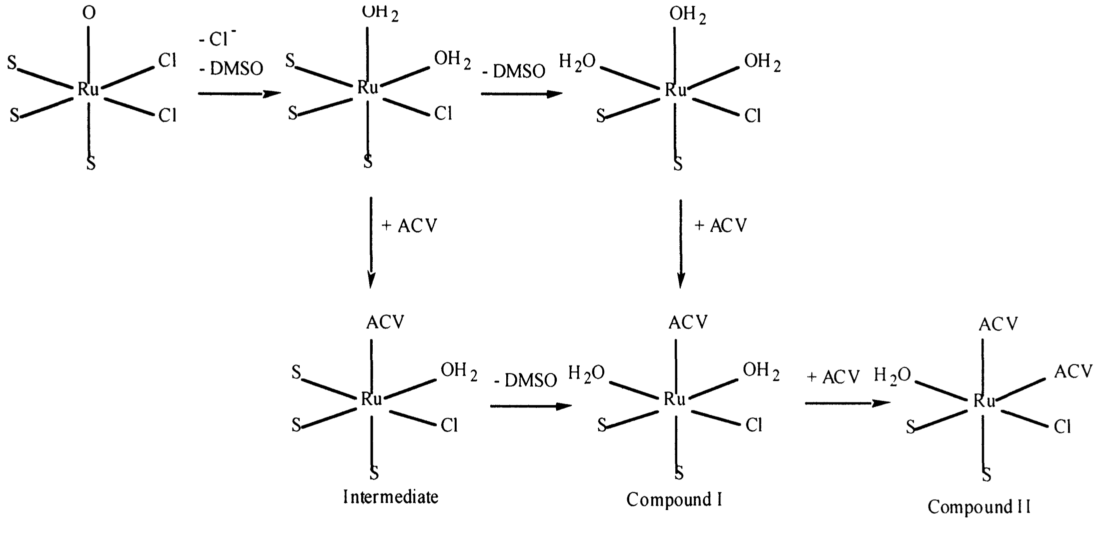

Scheme3. Proposed mechanism for the reaction of *cis*-[$RuCl_2(DMSO)_4$] with ACV in water solution. S = *S*-bonded DMSO, O = *O*-bonded DMSO. The overall charge of the complexes is not indicated, it is 0 for species containing two chlorine ligands and +1 for species containing only one chlorine ligand.

In the reaction of *cis*-Ru with 5'-GMP [29], the guanine N(7) and a phosphate oxygen both bind to Ru(II) and form a chelate with the metal center. Such a chelate does not form in the case of acyclovir due to the much poorer coordination ability of the hydroxyl group as compared to that of a phosphate. As a consequence the monoadduct reacts further with an acyclovir molecule to give the bisadduct. Similarly the major product in the reaction of *cis*-Ru with the dinucleotides GpA, ApG, d(ApG), and d(GpA) [21] was a disubstituted compound in which both purines of the dinucleotides were coordinated to the metal through the N(7) atoms.

The proposed reaction route for the *cis*-Ru isomer is depicted in Scheme 3.

The formation of complex I appears to be faster in the case of the *trans*-Ru complex as compared to that of the *cis* isomer. This might imply that in both cases the reactive species is the diaqua complex which is formed immediately after dissolution in water in the case of the *trans*-Ru complex, while it is formed more slowly in the case of the *cis*-Ru isomer. The conversion of the monoadduct into the bisadduct presumably takes place with the same rate in the two cases (this is true also in the light of the same identity of complexes I and II in the two cases). As a consequence in the case of *trans*-Ru there is accumulation of monoadduct in the initial stage of the reaction, afterwards the concentration of monoadduct decreases because it reacts with a second molecule of ACV to give the bisadduct. In the case of *cis*-Ru such an accumulation is not observed because of the smaller rate of formation of the monoadduct.

It is also to be noted that, substitution of one and two solvent molecules by ACV ligands in the diaqua species of *trans*-Ru and *cis*-Ru would lead to mono- and di-substituted species differing in the nature of the axial ligands (two chlorides in the former case and one chloride and one DMSO in the latter case). The obtainment of identical species in both cases implies that meanwhile the reaction progresses hydrolysis of one axial ligand ($Cl^-$ in *trans*-Ru and DMSO in *cis*-Ru) leads to identical species containing one chloride and one solvent molecule in axial positions. Such a solvolysis of an axial ligand has been taken into account in drawing Schemes 2 and 3. The slower rate of formation of the monoadduct in the *cis*-Ru complex also accounts for the relatively smaller concentration of monoadduct (with respect to the bisadduct) at the end of the reaction.

### CONCLUSIONS

The present investigation, apart from confirming the analogy in the coordination behavior of acyclovir with respect to other guanine bases, has also underlined some substantial differences between the behavior of antitumoral platinum(II) and ruthenium(II) complexes. These differences are: i) Greater propensity of the ruthenium(II) complexes to give the bis-adducts as compared to monoadducts. Under similar experimental conditions (e. g. use of an excess of metal substrate with respect to the purine ligand) platinum(II) substrates would have given, almost exclusively, the monoadduct. ii) Analogy in the behavior of the *cis* and the *trans* isomers of the ruthenium(II) complex which give exactly the same mono and bisadducts independently from the different coordination geometry of the starting substrate. This is not the case of platinum(II) species which give different reaction products depending upon the *cis* or *trans* geometry of the starting substrate. These results may be relevant to the different antitumor activity of platinum and the ruthenium substrates.

Although giving similar products, the rate of formation of the monoadduct is higher in the case of the *trans*-Ru complex as compared to that of the *cis*-Ru isomer. This might imply that in both cases the reactive species is the diaqua complex. This would also account for the greater propensity of the ruthenium substrates to give the bisadduct as compared to the platinum substrates.

An investigation of the antiviral and antitumoral activities of the ruthenium adducts with acyclovir (both the mono and the bis derivatives) is under way. It will be interesting to compare the results with those already obtained for the antiviral and anticancer activity of the platinum complexes with acyclovir.

### ACKNOWLEDGEMENTS

The authors like to thank the General Secretariat of Research and Technology (GSRT), Greece, and the Italian Ministry for Foreign Affairs, Italy, for the grant 13843/8.10.99 of the Greek-Italian bilateral agreement. G. N. also thanks the University of Bari (Contribution ex 60%), the Ministry for University and Scientific and Technological Research (MURST, Cofin. 1988 n° 9803021072) and the EC (COST Chemistry projects D8/0012/97 and D20/001/00.

### REFERENCES

1. *Cisplatin. Chemistry and Biochemistry of a leading Anticancer Drug* (Ed.: B. Lippert), Wiley VCH, Zurich, 1999.
2. G. Sava, S. Pacor, F. Bregant, V. Ceschia, G. Mestroni, *Anti-Cancer Drugs* **1**, 99 (1990).
3. G. Sava, S. Pacor, S. Zorzet, E. Alessio, G. Mestroni, *Pharmacol. Res.* **21**, 617 (1989).
4. M. Coluccia, G. Sava, F. Loseto, A. Nassi, A. Boccarelli, D. Giordano, E. Alessio, G. Mestroni, *Eur. J. Cancer* **29A**, 1873 (1993).
5. E. Alessio, G. Mestroni, G. Nardin, W.M. Attia, M. Calligaris, G. Sava, S. Zorzet, *Inorg. Chem.* **27**, 4099 (1988).
6. G. Mestroni, E. Alessio, G. Sava, S. Pacor, M. Coluccia, *Metal complexes in cancer chemotherapy*, K. Keppler (ed), VCH, Weinheim, 159 (1993).
7. S. Cauci, E. Alessio, G. Mestroni, F. Quadrifoglio, *Inorg. Chim. Acta* **137**,19 (1987).
8. G. Mestroni, E. Alessio, M. Calligaris, W. M. Attia, F. Quadrifoglio, S. Cauci, G. Sava, S. Zorzet, S. Pacor, C. Monti-Bragadin, M. Tamaro, L. Dolzani, *Prog. Clin. Biochem. Med.* **10**, 71 (1989).
9. F. Loseto, E. Alessio, G. Mestroni, G. Lacidogna, A. Nassi, D. Giordano, M. Coluccia, *Anticancer Res.* **11**, 1549 (1991).
10. G. Sava, A. Bergamo, *Int. J. Oncol.* **17**, 353 (2000).
11. L. Gavallo, R. Cini, J. Kobe, L. G. Marzilli, G. Natile, *J. Chem. Soc., Dalton Trans*, 1867 (1991).
12. S. Grabner, J. Plavec, N. Bukovec, D. Di Leo, R. Cini, G. Natile, *J. Chem. Soc. Dalton Trans*, 1447 (1998).
13. R. Cini, S. Grabner, N. Bukovec, L. Cerasino, G. Natile, *Eur. J. Inorg. Chem.* 1601 (2000).
14. M. Coluccia, A. Boccarelli, C. Cermelli, M. Portolani, G. Natile, *Metal-Based Drugs*, **2**, 249 (1995).
15. Z. Balcarova, J. Kasparkova, A. Zakovska, O. Novakova, M. F. Sivo, G. Natile, V. Brabec, *Mol. Pharmacol.* **53**, 846 (1998).
16. I.P. Evans, A. Spencer, G. Wilkinson, *J. Chem. Soc., Dalton Trans.* 204 (1973).
17. S. Cauci, P. Viglino, G. Esposito, F. Quadrifoglio, *J. Inorg. Biochem.* **43**, 739 (1991).
18. J. M. Davey, K. L. Moerman, S. F. Ralph, R. Kanitz, M. M. Sheil, *Inorg. Chim. Acta* **281**, 10 (1998).
19. E. Alessio, Y. Xu, S. Cauci, G. Mestroni, F. Quadrifoglio, P. Viglino, L. G. Marzilli, *J. Am. Chem. Soc.* **111**, 7068 (1989).
20. G. Esposito, S. Cauci, F. Fogolari, E. Alessio, M. Scocchi, F. Quadrifoglio, P. Viglino, *Biochemistry* **31**, 7094 (1992).
21. A. Anagnostopoulou, E. Moldrheim, N. Katsaros, E. Sletten, *J. Biol. Inorg. Chem.* **4**, 199 (1999).
22. W. S. Sheldrick, S. Heeb, , *Inorg. Chim. Acta* **168**, 93 (1990).
23. P. M. van Vliet, J. G. Haasnoot, J. Reedijk, *Inorg. Chem.* **33**, 1934 (1994).
24. B. Blazic, N. Bukovec, P. Bukovec, F. Lazarini, *Z. Kristal.* **185**, 355 (1988).
25. I. Turel, N. Bukovec, M. Goodgame, D. J. Williams, *Polyhedron* **16**, 1701 (1997).
26. I. Turel, B. Andersen, E. Sletten, A. J. P. White, D. J. Williams, *Polyhedron* **17**, 4195 (1998).
27. A. Sinur, S. Grabner, *Acta Cryst.* **C51**, 1769 (1995).
28. A. Garcia-Raso, J. J. Fiol, F. Badenas, R. Cons, A. Terron, M. Quiros, *J. Chem. Soc. Dalton Trans*, 167 (1999).
29. Yan-Ni tian, Pin Yang, Qing-Shan Li, Mao-Lin Guo, Ming-Gen Zhao, *Polyhedron* **16**, 1993 (1997).

**Received: January 11, 2001 - Accepted: January 21, 2001 - Received in revised camera-ready format: January 22, 2001**

